# Esmarch Bandage and Its Substitutes

**Published:** 1876-08

**Authors:** R. J. Levis

**Affiliations:** Surgeon to the Pennsylvania Hospital


### The Esmarch Bandage and its Substitutes.

By R. J. Levis, M. D., Surgeon to the Pennsylvania Hospital.

The advantage of the elastic bandage in securing bloodless operations are now generally recognized by surgeons, and some practical suggestions, intended to impress the profession in regard to its great merits and which will facilitate its general use, may be acceptable.

In the last issue of the *American Journal of the Medical Sciences*

is a communication from Dr. David Little, which is inclined to disparage the merits of the elastic bandage, as compared with the substitution of an ordinary muslin roller bandage which he recommends for such purposes. Immediately on the first publication of Professor Esmarch's article on the avoidance of hemorrhage by the use of an elastic bandage, and before the proper appliance was obtainable, I repeatedly used the ordinary muslin roller to effect the compression, and am able, from practical experience, to give evidence of its decided inferiority to the india-rubber bandage.

The peculiar advantage of the elastic bandage is the *equable* and *continuing* pressure which is produced in applying it, so that the blood is gradually and continuously forced upward, and none is retained, as with the muslin bandage, between irregularly constricting lines of the circular turns. The muslin roller well applied may answer somewhat effectively, as I know from experience, particularly on attenuated limbs or on the distal portions of extremities; but surgeons may be seriously disappointed in producing with it bloodless operations in the upper portions of limbs of vigorous patients, especially of those which are large or very fat. If, on an emergency, the simple muslin bandage be ever resorted to, I recommend that two such bandages be placed on the limb, every turn of the bandage made by the surgeon being *immediately* followed by another similar bandage in the hands of an assistant; thus equable, continuous, and forcible pressure may be effected. A better and more effective bandage than that of muslin for bloodless operating may be made of a good quality of ordinary flannel, long enough to encase the limb well, with full allowance for overlapping of edges.

I have for a long time discarded the use of the elastic webbing and constricting tubing, as proposed by Professor Esmarch, in favor of a bandage made of pure india-rubber. The bandage which I am in the habit of encasing the limb is five yards long and two and a half inches wide; and the constricting band or tourniquet is of a much thicker and stronger material, two yards in length and one inch and three-quarters in width. The pure india-rubber bandage has the merits of greater elasticity, strength, durability, cleanliness, cheapness, and more perfect adaptability. The constricting band is perfectly effective as a tourniquet, without the great disadvantage of the narrow tubing of Professor Esmarch in producing severe linear pressure, which, it is known, has often caused more or less enduring paralysis from extreme nerve-compression. My tourniquet band is without the complications of chain and hook, as in Professor Esmarch's, the final securing being simply effected by tying together pieces of strong cord, one of which is permanently attached through a hole in each end. The elastic bandage, as I have described it, can be procured of Mr. Levick, manufacturer of india-rubber articles, 724 Chestnut street, Philadelphia.

It should be borne in mind that the advantage of the Esmarch bandage is not alone in the avoidance of the loss of blood in the patient, but every surgeon who has effectively used it in operations as the excisions of joints or of necrosed bone is aware of how the completely exsanguinous condition of the tissues, resembling those of the cadaver, facilitates the accuracy of the procedure.

Mr. Erichsen has made the remark that he "fears that the cultivation of surgery as an *art* is not keeping pace with the progress of surgery as a *science;*" and the surgeon who appreciates the value of what is well expressed in an aphorism from a recent address of Sir William Fergusson, that "the glory of surgery is *precision,*" will surely recognize in the use of the elastic bandage an advancement in the precision of surgical art.—*Phila. Med. Times.*

